# Phosphorylated proteomics analysis of human coronary artery endothelial cells stimulated by Kawasaki disease patients serum

**DOI:** 10.1186/s12872-018-0982-2

**Published:** 2019-01-17

**Authors:** Shui-Ming Li, Wan-Ting Liu, Fang Yang, Qi-Jian Yi, Shuai Zhang, Hong-Ling Jia

**Affiliations:** 1 College of Life Sciences and Oceanography, Shenzhen Key Laboratory of Microbial Genetic Engineering, Shenzhen University, Shenzhen, Guangdong China; 2 Key Laboratory of Functional Protein Research of Guangdong Higher Education Institutes, Institute of Life and Health Engineering, College of Life Science and Technology, Jinan University, No.601, West Huangpu Avenue, Guangzhou, 510632 Guangdong China; 3 Department of Pediatrics, First Affiliated Hospital of Jinan University, Guangzhou, China; 4 Department of Medical Biochemistry and Molecular Biology, School of Basic Medical Sciences, Jinan University, Guangzhou, Guangdong China; 5 Department of Cardiovascular Medicine, Children’s Hospital of Chongqing Medical University, Ministry of Education Key Laboratory of Child development and Disorder, China International Science and Technology Coorperation base of Child development and Critical Disorder, Chongqing Key Laboratory of Pediatrics, Chongqing, China

**Keywords:** KD, HCAECs, Phosphorylated proteomics, Network analyzer analysis, Hub proteins

## Abstract

**Background:**

Kawasaki disease (KD) is an acute febrile childhood systemic vasculitis that disturbs coronary arteries. The pathogenesis remains unknown. The study of phosphorylated proteins helps to elucidate the relevant pathophysiological mechanisms of cardiovascular disease. However, few researches explored phosphorylated proteins in KD patients.

**Methods:**

We compared phosphoprotein profiles of HCAECs stimulated by the serum of KD patients and normal children using iTRAQ technology, TiO_2_ enrichment phosphorylated peptide and MS analysis. Then we conducted the functional analysis by ClueGO and the biological interaction networking analysis by ReactomeFIViz. Western blotting was performed to identify the hub proteins.

**Results:**

Our results revealed that phosphorylation of 148 proteins showed different intensities between the two HCAECs groups, which are enriched in MAPK, VEGFR, EGFR, Angiopoietin receptor, mTOR, FAK signaling pathway and so on. Through the Network Analyzer analysis, the hub proteins are CDKN1A, MAPK1 and POLR2A, which were experimentally validated.

**Conclusion:**

In summary, we provided evidence addressing the valuable phosphorylation signaling that could be useful resource to understand the molecular mechanism and the potential targets for novel therapy of KD.

**Electronic supplementary material:**

The online version of this article (10.1186/s12872-018-0982-2) contains supplementary material, which is available to authorized users.

## Background

Kawasaki disease (KD) is an immune-related multisystem vasculitis and usually occurs in children under 5 years of age, but whose etiology remains unknown. As the first pathogeny of acquired heart disease instead of rheumatic fever, KD is a novel risk factor of forming atherosclerosis and ischemic heart disease that have been reported by more and more previous studies. The acute vasculitis associated KD may lead to the development of a complex set of coronary artery abnormalities including coronary artery dilatation and coronary aneurysm. And coronary artery stenosis or thrombosis, or even myocardial infarction could occur in later period. However, the pathomechanism of coronary artery abnormalities complicated with KD is still not well understood, making specific molecular diagnosis and therapy thus far impossible.

Protein phosphorylation is one of the most basic, common and important mechanisms for regulating protein activation and function. A variety of biological processes are closely related to the protein phosphorylation which play on/off regulatory role for many biochemical functions, such as transcriptional and translational regulation, signal transduction, DNA damage repair, cell metabolism, secretion, homeostasis and so on. Abnormal phosphorylation associated with many diseases, and the production of phosphorylation is as the footprints of the abnormal status which exists in organisms. Therefore, the study of phosphorylated proteins in biological samples helps to elucidate the relevant pathophysiological mechanisms. Many studies have reported that phosphorylation of proteins play an important regulatory role in the occurrence and development of cardiovascular disease [1, 2]. Some protein kinases affect the structure and function of vascular smooth muscle cells by mutual regulation with ANG II, PDGF, ET-1 and other vasoactive substances, and therefore directly involved in the pathological process of cardiovascular disease. There have been some previous proteomics studies on KD in serum and urine [3–5]. However, there are few protein phosphorylation studies in field of KD.

Hence, this study focused on KD protein phosphorylation to identify differentially expressed phosphoproteins and phosphorylated molecules in KD by large-scale high-throughput approaches: the isobaric tags for relative and absolute quantitation (iTRAQ) technology, titanium dioxide(TiO_2_) enrichment phosphorylated peptides, mass spectrometry(MS) analysis [6] to investigate the phosphoproteome analysis of KD patients, and revealed that the phosphoprotein plays an important role in the pathogenesis of KD. The study may provide an insightful understanding of KD precise pathomechanism and implications for the therapy of KD in phosphorylation protein level.

## Methods

### Collection of serum samples of KD patients and healthy children

Ethical approval was obtained for children with KD and healthy children clinical sample collection from the Ethics Committee at First Affiliated Hospital of Jinan University and written informed consent was obtained from the guardians of all children. Serum samples from children with KD diagnosed were randomly selected according to the revised digest version of guidelines from the Japanese Circulation Society Joint Working Groups performed in 2012 [7]. The information of the children with KD is shown in Table 1. Serum samples from sex and age matched normal children were used as the control group. Serum aliquots were collected and stored at − 80 °C refrigerator.

**Table 1:** Clinical indicators of 8 KD patients

Patient	Age-ranges	Gender	Coronary Change
Patient 1	2-5Y	male	LCA = 3.0 mm, RCA = 2.6 mm.
Patient 2	1–2 Y	male	LCA = 2.8 mm, RCA = 2.5 mm.
Patient 3	2-5Y	male	LCA = 4.0 mm, RCA = 3.6 mm.
Patient 4	2-5Y	female	LCA = 3.0 mm, RCA = 2.5 mm.
Patient 5	2-5Y	male	LCA = 3.5 mm, RCA = 3.6 mm.
Patient 6	2-5Y	male	LCA = 3.2 mm, RCA = 2.8 mm.
Patient 7	2-5Y	female	LCA = 3.2 mm, RCA = 3.0 mm.
Patient 8	1–2 Y	female	LCA = 3.2 mm, RCA = 3.3 mm.

*Y*: year; *LCA*: left coronary artery; *RCA*: right coronary artery

### Human coronary artery endothelial cells (HCAECs) culture and preparation

HCAECs (human coronary artery endothelial cells) were obtained from ScienCell (Carlsbad, CA, USA) and cultured using an endothelial cell growth medium containing growth factors, supplements and 10% fetal bovine serum. When HCAECs were 90% confluent, the medium was exchanged to endothelial cell basal medium and the cells were incubated with 15% serum from KD patients or healthy children respectively. After culturing of HCAECs for 24 h, two HCAECs groups (Control and KD) were collected.

### Protein preparation and iTRAQ labeling

Control and KD groups were washed three times with ice-cold washing buffer (10 μM Tris-HCl, 250 μM sucrose, pH 7.0) and transferred to a clean 1.5 ml Eppendorf tube. Cells were lysed with a buffer containing 7 M urea, 2 M thiourea, 4% CHAPS, 0.2 mg/ml PMSF, phosphatase inhibitors cocktail (Roche, Basel, Switzerland) and protease inhibitors (Roche, Basel, Switzerland). Cellular debris was removed by centrifugation for 30 min at 13,000 g and at 4 °C. Protein concentration was determined by Protein Assay Kit. The total proteins of each group were analyzed by iTRAQ-based liquid chromatography and tandem mass spectrometry/mass spectrometry (LC-MS/MS). 200 μg protein sample from each group was reduced, alkylated, and subjected to tryptic hydrolysis [8]. ITRAQ labeling was performed on the basis of iTRAQ Regents manufacturer’s protocol. Each sample was labeled separately with the iTRAQ tags as follows: KD group was labeled with 113/115 isobaric tags;Control group was labeled with 117/119 isobaric tags separately, and then all labeled peptides were pooled and evaporated to dryness in a vacuum centrifuge.

### High-pH reversed-phase liquid chromatography

Firstly, iTRAQ labeled samples were diluted to 100 μl with H_2_O buffer (NH_3_•H_2_O, pH = 10) before high performance liquid chromatography (HPLC) on a Gemini-NX 3u C18 110A; 150× 2.00 mm Phenomenex columns and Gemini 3u C6-Phenyl 110A; 100 × 2.0 mm. The flow rate with 0.2 ml/min was used for reversed-phase column separation by H_2_O (mobile phase A) and 80% acetonitrile (ACN) (mobile phase B). A solvent gradient system was used as described previously [8].

### Phosphopeptides enrichment employing TiO_2_ resin

Phosphopeptide Enrichment TiO_2_ kit (Calbiochem, San Diego, CA, USA) was used to enrich the phosphopeptides after peptide digestion according to the manufacturer’s instruction with minor modifications. In brief, the production of trypsin digestion was dried and redissolved in 200 μl TiO_2_ phosphobind buffer with 50 g/L 2, 5-dihydroxybenzoic acid. Then 50 μl TiO_2_ phosphobind resin was added and incubated for 30 min. After discarding supernatant, TiO_2_ was rinsed for three times with the wash buffer. Elute the phosphopeptides with elution buffer for twice and combine all the eluates. And then the eluates were dried using a Speed-Vac concentrator and reconstituted in 2% ACN/1% TFA for LC-MS/MS analysis.

### Peptides analysis by the LC-MS/MS approach

Dried phosphopeptides were analyzed with a Triple TOF 5600 plus nano ESI-LCMS instrument. Briefly, the peptide mixtures were loaded in a C18 column (5 μm resin from Michrom Bioresources, 10 cm long, 100 μm i.d., Auburn, CA, USA) using an autosampler. Peptides were eluted by the 0–35% gradient buffer solution (Buffer A: 5% ACN and 0.1% formic acid; Buffer B: 95%ACN and 0.1% formic acid) for more than 90 min and subsequently online detected in the Triple TOF 5600 plus mass spectrometer using an information dependent acquition mode (IDA) method which allows top 20 precusor ions selected for MS/MS analysis in each cycle. The general mass spectrometric conditions were as the follows: spray voltage of 2.3 kV; curtain gas of 35 psi, nebulizer gas of 5 psi, and an interface heat temperature of 150 °C.The MS and MS2 analysis was operated in positive TOF-MS and production ion scan mode respectively. For IDA analysis, survey scans were acquired in 250 ms and as many as 20 product ion scans (80 ms) were collected if the precursor ion intensity passed the threshold of 200 cps with the charge state of between of + 2 to + 5. A rolling collision energy setting was applied to all precursor ions. Dynamic exlusion was set for 16 s.

### Database analysis and manual evaluation of mass spectra

The MS/MS data were analyzed for protein identification and quantification using ProteinPilot Software v.4.5 (AB Sciex, Framingham, MA, USA). The local false discovery rate was estimated with the integrated PSPEP tool in the ProteinPilot software to be 1.0% after searching against a decoy concatenated uniprot human protein database (20,210 protein entries). The database search parameters were as the followings: iTRAQ 8-plex quantification, cysteine modified with iodoacetamide, phosphorylation emphasis, trypsin digestion, thorough searching mode and minimum protein threshold of 95% confidence (unused protein score > 1.3). The same raw files used for protein pilot 4.5 search analysis were further exported into MGF format peak list files and then submitted to mascot search engineer for protein identification and relative quantitation analysis. The search parameters were the same as those employed in proteinpilot software. Mass spectra of identified phosphopeptides with confidence between 90 and 95% should also meet the following criteria: two consecutive b- and/or y-ion series and extensive coverage of b- and/or y-ion series were required, an obvious observation of neutral loss of 98 Da of the precusors.

### Western blot analysis

After lysis, the protein samples of two groups were separated by 12% SDS-PAGE, electrophoresed and transferred onto a PVDF membrane. Blots were detected with several antibodies CDKN1A (cyclin-dependent kinase inhibitor 1A), MAPK1 (Mitogen-activated protein kinase-1, also known as ERK2 or p42 MAPK), POLR2A (DNA-directed RNA polymerase II subunit RPB1), phospho(p)-CDKN1A (Thr145), p-MAPK1 (Thr202/Tyr204), p-POLR2A (Ser1619) following the standard protocol. CDKN1A, MAPK1, p-CDKN1A (Thr145) and p-MAPK1 (Thr202/Tyr204) antibodies were purchased from Abcam Inc., USA and other antibodies including POLR2A and p-POLR2A (Ser1619) were purchased from Novus Biologicals Inc., USA. All immunoblot detections were performed with horseradish peroxidase-conjugated secondary antibodies and chemiluminescence detection system. The quantitative analysis of band intensities was performed using Photoshop software.

### Protein categorization and protein-protein interaction network constructions

The Cytoscape plugin GlueGO and ReactomeFIViz were applied for the differentially expressed phosphoproteins to process the biological process of GO term and KEGG pathway enrichment analysis. The *p*-value< 0.05 and FDR < 0.01 were as threshold values for the GO and KEGG pathway enrichment. ReactomeFIViz also provides the associations of these differential expressed proteins which were visualized by network illustration. Then, the betweenness centrality and the number of direct edges were analyzed by Cytoscape plug-in tool ‘Network Analyzer’, and the analysis results were showed by progressive changed node sizes and colors.

## Results

### Quantitative phosphoproteomics analysis of HCAECs of KD

To obtain a global view change of protein phosphorylation in KD, we simulated HCAECs with serum from KD patients to mimic microenvironment of KD [9–11]. All MS/MS spectra were respectively searched, against the reversed and forward human protein sequence database to estimate rates of false-positive matches after LC-MS/MS analysis on the enriched phosphopeptides. The 5929 phosphopeptides from the target database passed our criteria. The false positive rate of phosphopeptide was hence estimated to be 1.3%. Multiple filtering standard were established to test and verify search results. For each of the identified phosphorylated peptides in this work, peptide sequences were manually confirmed. We detected 238 unique phosphorylation sites from 233 unique phosphopeptides corresponding to 148 protein groups including the distributions of phosphorylated threonine, serine, and tyrosine sites, respectively, after these validations. This whole dataset was provided as Additional file 1: Table S1. These results are in line with those from a previous study on a variety of cell types: the overall level of phosphotyrosine in proteins was very low compared to the level of phosphoserine and phosphothreonine in HCAECs of KD.

### Functional analysis of identified phosphoproteins of HCAECs of KD

**Fig. 1:**
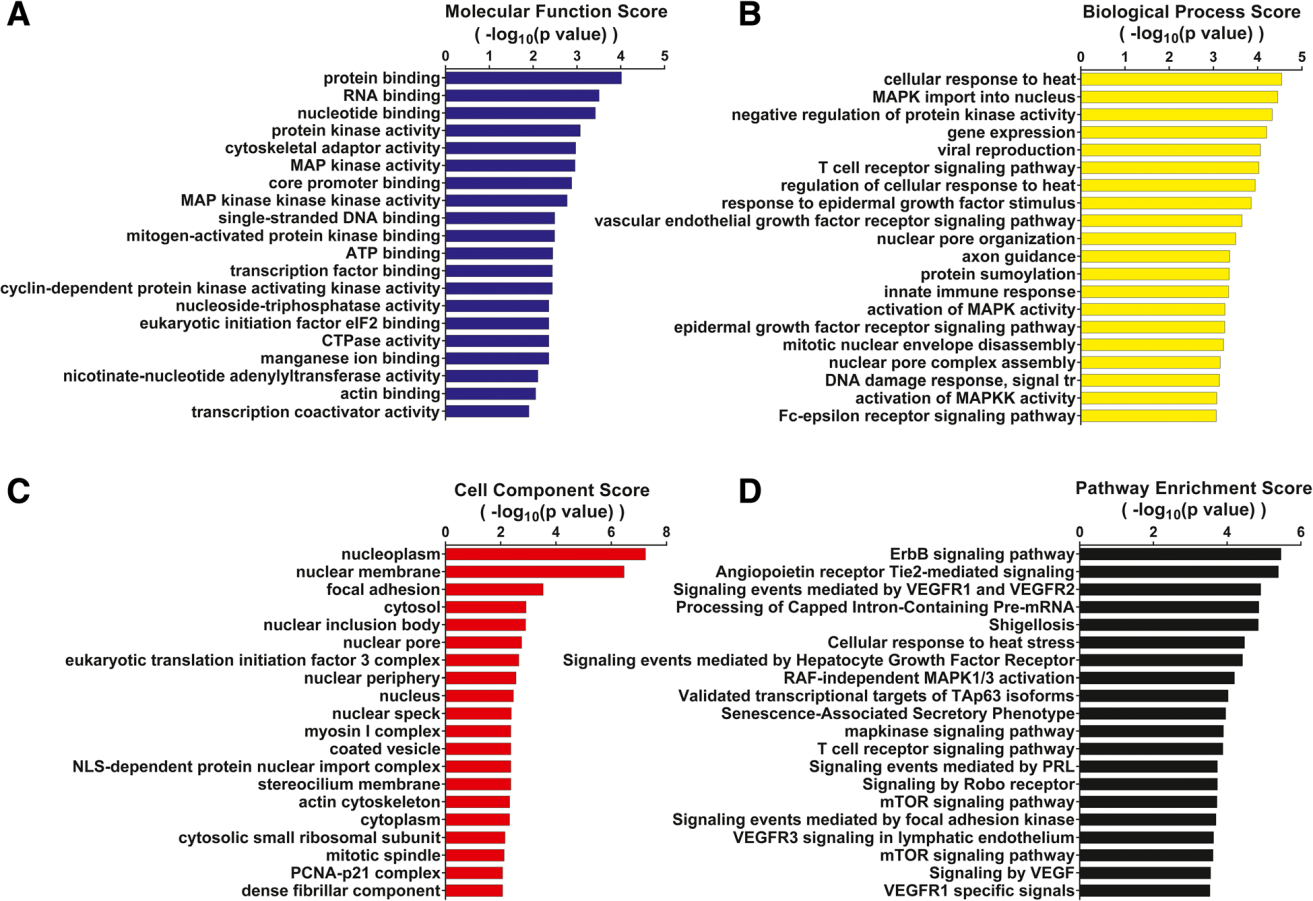
Categorization of the differentially expressed phosphoproteins of HCAECs in KD. **a** The top 20 molecular function identified. **b** The top 20 biological processes identified. **c**The top 20 cell compoment identified. **d** The top 20 pathway enrichment analysed by Cytoscape in HCAECs simulated KD patient serum. The log-transformed enrichment scores for each molecular function, biological process, cell compoment and pathway are indicated on the x-axis

In order to understand the biological relevance of phosphoproteins, the molecular functions, biological processes and protein classification of the differentially expressed proteins were analysed using ClueGO that is a Cytoscape plug in software, which integrates gene ontology terms. Bar charts were used to show the distribution of biological process, functional categories for these differentially expressed proteins of HCAECs in KD. Figure 1 provides an overview of KD phosphoproteome based on the known or postulated functions or biological processes of the identified phosphoproteins.

### Biological interaction networking of identified phosphoproteins

**Fig. 2:**
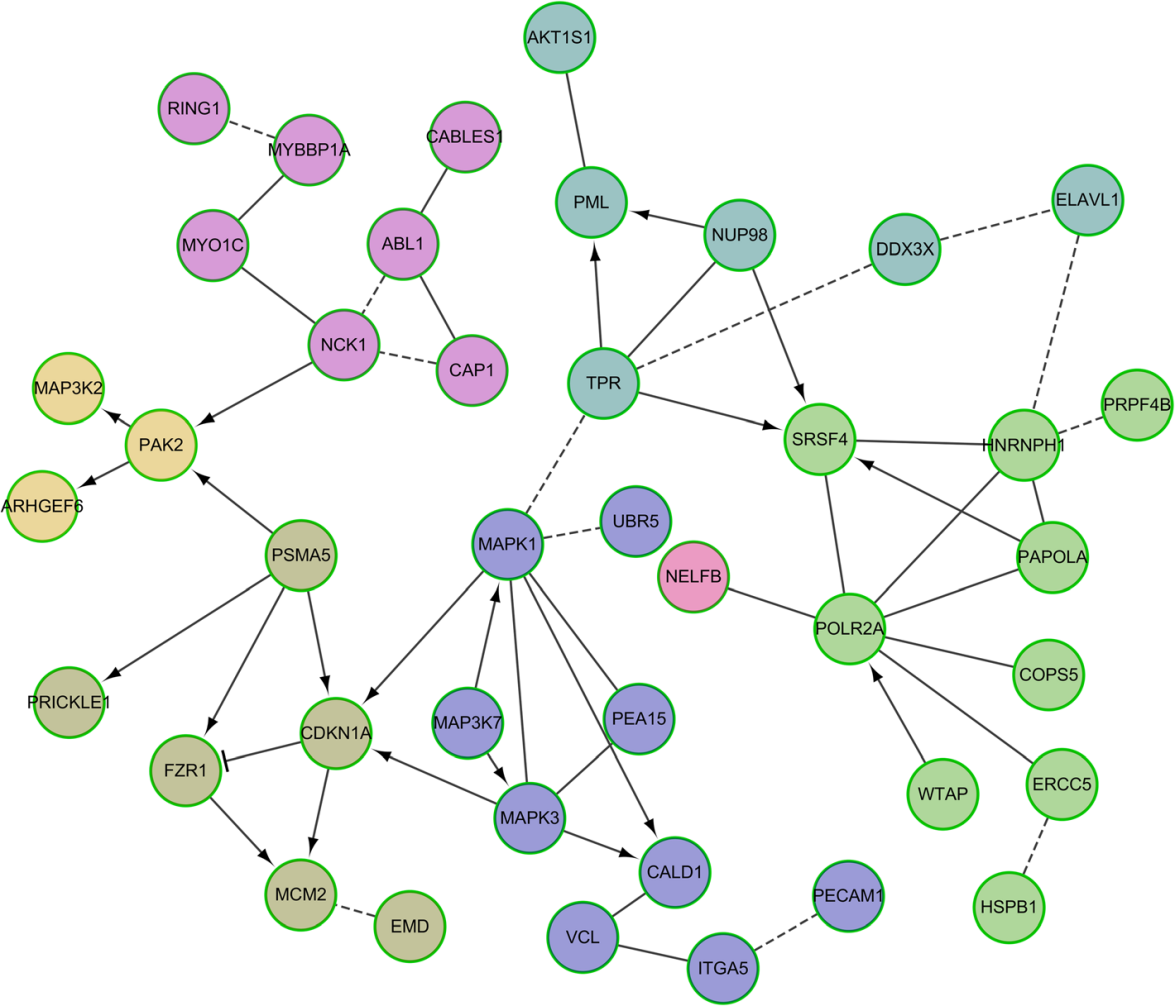
Biological interaction networking of identified HCAECs phosphoproteins in KD. Proteins of the network were differentially expressed phosphoproteins of HCAECs of KD which were functionally enriched based on KEGG pathway

**Fig. 3:**
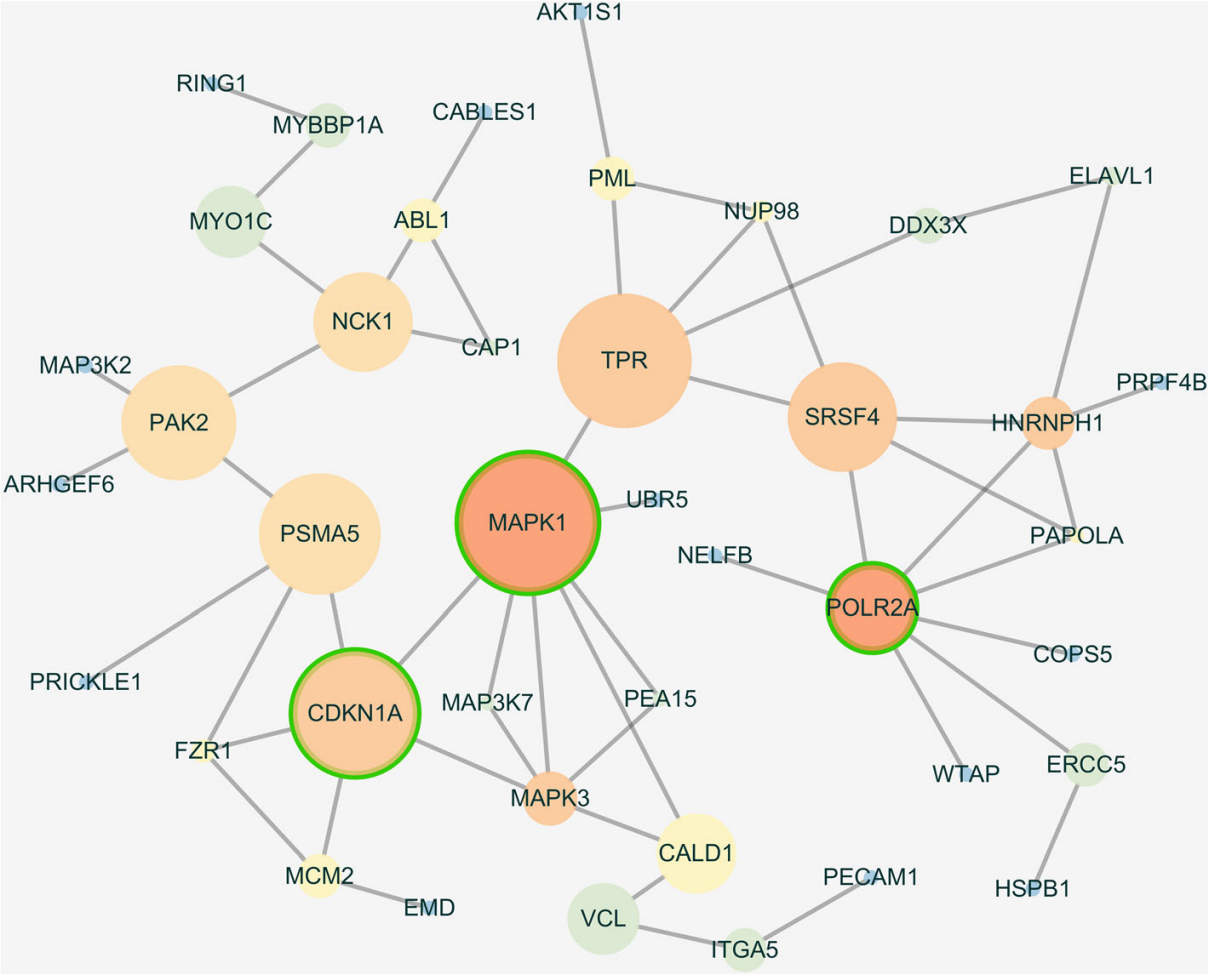
The outcome of network analysis. The node sizes showed the results of betweenness centrality analysis. The node colour was decided by the numbers of direct edges to indicate the important of the node. The node with green circle demonstrated the proteins were selected for western blotting verification because they contained the best results of the analysis

To comprehensively characterize the relationships among significantly differentially expressed phosphoproteins of HCAECs in KD, we were subjected to the KEGG pathway enrichment analysis and signalling network modelling by the Cytoscape plugin ReactomeFIViz. The thresholds of pathway enrichment were defined as *p* < 0.05, FDR < 1%. We constructed a biological interaction networking of the phosphoproteins identified of HCAECs in KD. The phosphoproteins that could be networked were linked by various relationships such as protein-protein interactions, modifications and regulation of expression. The network was deeply analyzed by Network Analyzer which is a Cytoscape plug-in tool for betweenness centrality and number of direct edges shown in Figs. 2 and 3, the progress changed colours and node sizes showed which nodes play core roles in the whole network, viz. the more betweenness the bigger of node sizes, the more number of direct edges the darker red colour. In other words, the node with the deepest red and the biggest size was the most important core. According to this rule, CDKN1A, MAPK1 and POLR2A were selected.

### Validation of differential expressed phosphoproteins

**Fig. 4:**
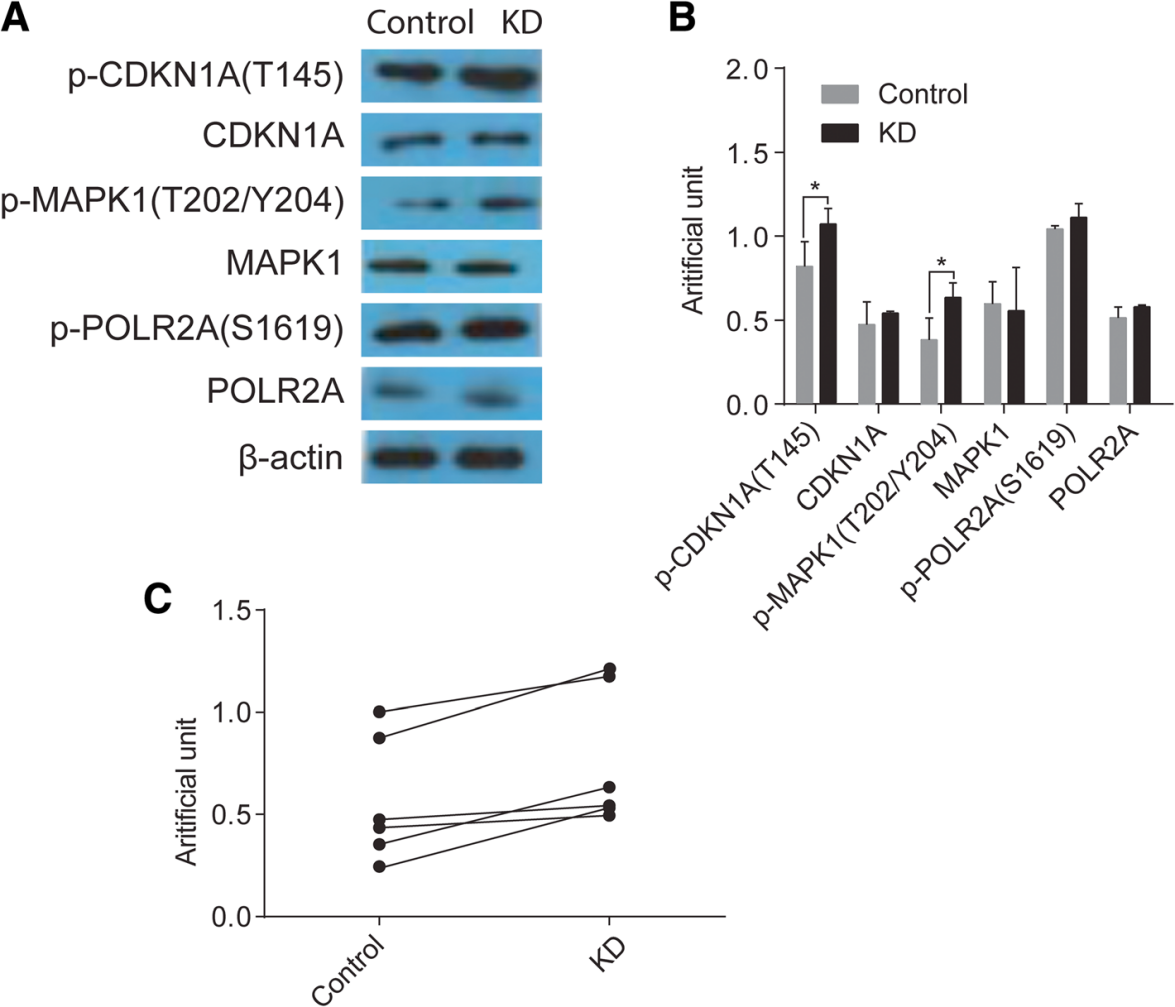
Validation of phosphorylated proteomics results by Western blot. **a** Western blots of CDKN1A, MAPK1, POLR2A, p-CDKN1A, p-MAPK1, p-POLR2A of HCAECs in KD, β-actin was used as the internal control. **b** Statistical analysis of the band intensities in A. **c** Grouped analysis of the band intensities of A. Data represent mean ± SD. Statistical significance is determined by Student’s *t* test, *p* < 0.05

To further confirm the results from the quantitative phosphoproteome analysis, we chose CDKN1A, MAPK1, POLR2A, p-CDKN1A, p-MAPK1, p-POLR2A, for Western blotting verification using an anti-CDKN1A antibody, anti-MAPK1 antibody, anti-POLR2A antibody, anti-p-CDKN1A (Thr145) antibody, anti-p-MAPK1 (Thr202/Tyr204) antibody and anti-p-POLR2A (Ser1619) antibody. As shown in Fig. 4, the results of Western blotting were consistent with phosphoproteomics analysis for these differentially expressed proteins. Phosphorylation of CDKN1A, MAPK1 and POLR2A are significantly increased in HCAECs of KD, whereas steady-state CDKN1A, MAPK1 and POLR2A remained almost unchanged in Western blotting verification.

## Discussion

To date, little study has been carried out on the phosphoproteome in KD. To explore pathophysiology-related molecules from the aspect of phosphorylation in KD, we compared phosphoprotein profiles of HCAECs of KD and normal children. Among the detected 5929 phosphopeptides, 233 phosphopeptides corresponding to 148 protein groups showed different intensities between the two HCAECs groups. The identified differentially expressed phosphoproteins may be used as potential biomarkers to facilitate KD diagnosis and monitoring of treatment effectiveness and it would reflect a deeper understanding of pathological processes of coronary artery abnormalities complicated with KD.

Applying various analysis software tools, we identify several differentially regulated signaling pathways, such as MAPK (mitogen-activated protein kinase), VEGFR (vascular endothelial growth factor receptor), EGFR (epidermal growth factor receptor), Angiopoietin receptor, HGFR (hepatocyte growth factor receptor), mTOR (mammalian target of rapamycin), FAK (focal adhesion kinase), PRL signaling pathway. Some previous study have revealed that AMPK-mTOR and MAPK-ERK1/2 signaling pathway are involved in human vascular smooth muscle cells proliferation, which plays a key role in the pathogenesis of vascular diseases such as hypertension and restenosis [12–15]. In addition, FAK signaling and enhanced tyrosine phosphorylation is important for the human coronary artery smooth muscle cells and cardiac microvascular endothelial cells migration, which is the key process in the pathophysiology of restenosis and atherosclerosis [16, 17]. Previous studies have noted that the activated FAK, ERK, JNK, PI3K and AKT may promote angiogenesis and arteriogenesis, which is reported to be the mature form of new vessels and lead to an efficient restoration of blood flow [18].

Strikingly for this study, phosphopeptides from proteins including CDKN1A, MAPK1 and POLR2A were remarkably increased expression in KD. CDKN1A (also known as p21) regulates various biological activities by binding to and inhibiting the kinase activity of the CDKs (cyclin-dependent kinases, CDK2 and CDK1 also known as CDC2) leading to cell cycle arrest at specifics tags. Extensive reports in biochemistry and genetics shows that p21 is identified as an oncogene or tumor suppressor due to its up-regulation or down-regulation in several cancers [19, 20]. In addition, p21 stimulates cell proliferation of endothelial cell depending on attenuating CDK2 inhibition which is mediated by AKT1- phosphorylated p21 at T145 [21]. Furthermore, the phosphorylation of p21 by AKT1 in endothelial cells may have a role in promoting neovascularization and metastasis. Interestingly, our results showed that p-p21 (T145) was enhanced in KD, suggested that p21 phosphorylation may have an important role in coronary artery abnormalities of KD.

We are also specifically interested in MAPK1 that plays a pivotal role in cell development, proliferation, differentiation, transcription regulation. The activation of MAPK1 requires its phosphorylation on Tyr and Thr residues by upstream kinases, such as MEK2. Upon activation, MAPK1 translocates to the nucleus of the stimulated cells, where it phosphorylates nuclear targets. The previous studies have discovered that the ERK1/2 activation is able to protect cardiomyocytes against apoptosis [22]. Furthermore, the phosphorylation of MAPK1 can increase the proliferation of cardiomyocytes via up-regulating the expression of MALAT1 through PI3K/AKT signaling pathway [23]. The MAPK1 genetic mutations were speculated to be potential risk factors for heart defects, such as coronary artery disease considering hereditary variation among diverse ethnicities [24, 25]. Miura et al. reported the effect of MAPK activation on HDL-mediated signal transduction and angiogenesis induction in HCAECs [26]. And Plasma C-reactive protein, a prototypic marker of inflammation, regulated the expression of receptor for advanced glycation end-products via activation of the ERK/NF-κB signaling pathway in HCAECs [27]. However, there were few reports about the research on MAPK1 in coronary artery lesion of KD. Our data suggested that MAPK1 is probably important target of signaling pathways for the development and progression of KD.

The third protein we specifically considered is POLR2A (also known as RPB1), which contains a carboxy terminal domain composed of heptapeptide repeats that are essential for polymerase activity. These repeats contain serine and threonine residues that are phosphorylated in actively transcribing RNA polymerase. POLR2A gene located in close proximity to the tumor suppressor gene p53, which frequently shows loss of heterozyosity in cancer cells. Jesper V. Olsen et al. found that the kinase CDK7 phosphorylates POLR2A and regulates epithelial ovarian cancer cell proliferation in order to reveal a druggable kinase signature in ovarian cancer by phosphoproteomics [28]. Change of POLR2A phosphorylation suggest that dysregulation of RNA polymerase may be associated with KD occurrence and development.

## Conclusions

In sum, the in vivo quantitative phosphoproteomics from KD patient serum stimulated HCAECs could lead to provide valuable clues for decreasing the incidence of coronary artery lesions and improving the prognosis in KD. The study may provide an insightful understanding of KD precise pathomechanism and implications for the treatment of KD in protein phosphorylation level.

## Additional file

Additional file 1: **Table S1.** Phosphorylated proteins identified in HCAECs of KD, The entire dataset of 233 identified unique phosphopeptides. (XLSX 37 kb)

## Abbreviations

CDKN1A: Cyclin-dependent kinase inhibitor 1A
EGFR: Epidermal growth factor receptor
FAK: Focal adhesion kinase
HCAECs: Human coronary artery endothelial cells
HGFR: Hepatocyte growth factor receptor
iTRAQ: isobaric tags for relative and absolute quantitation
KD: Kawasaki disease
MAPK1: Mitogen-activated protein kinase-1
MS: Mass spectrometry
mTOR: Mammalian target of rapamycin
POLR2A RNA: Polymerase II subunit A
TiO_2_: Titanium dioxide
VEGFR: Vascular endothelial growth factor receptor
